# Health disparities monitoring in the U.S.: lessons for monitoring efforts in Israel and other countries

**DOI:** 10.1186/s13584-018-0208-1

**Published:** 2018-02-28

**Authors:** Kathleen Abu-Saad, Shlomit Avni, Ofra Kalter-Leibovici

**Affiliations:** 1 Gertner Institute for Epidemiology and Health Policy Research, Sheba Medical Center, Ramat Gan, Israel; 2 Strategic and Economic Planning Administration, Israel Ministry of Health, Jerusalem, Israel

**Keywords:** Health disparities monitoring, Health disparity measures, Social determinants of health, United States, Israel

## Abstract

**Background:**

Health disparities are a persistent problem in many high-income countries. Health policymakers recognize the need to develop systematic methods for documenting and tracking these disparities in order to reduce them. The experience of the U.S., which has a well-established health disparities monitoring infrastructure, provides useful insights for other countries.

**Main body:**

This article provides an in-depth review of health disparities monitoring in the U.S. Lessons of potential relevance for other countries include: 1) the integration of health disparities monitoring in population health surveillance, 2) the role of political commitment, 3) use of monitoring as a feedback loop to inform future directions, 4) use of monitoring to identify data gaps, 5) development of extensive cross-departmental cooperation, and 6) exploitation of digital tools for monitoring and reporting. Using Israel as a case in point, we provide a brief overview of the healthcare and health disparities landscape in Israel, and examine how the lessons from the U.S. experience might be applied in the Israeli context.

**Conclusion:**

The U.S. model of health disparities monitoring provides useful lessons for other countries with respect to documentation of health disparities and tracking of progress made towards their elimination. Given the persistence of health disparities both in the U.S. and Israel, there is a need for monitoring systems to expand beyond individual- and healthcare system-level factors, to incorporate social and environmental determinants of health as health indicators/outcomes.

**Electronic supplementary material:**

The online version of this article (10.1186/s13584-018-0208-1) contains supplementary material, which is available to authorized users.

## Background

Health disparities or inequities are defined as “avoidable and unjust differences in exposure and vulnerability to health risk factors, health-care outcomes and the social and economic consequences of these outcomes” ([1]: p 15). The reduction/elimination of health disparities has been a public health priority for the past several decades; with the recognition, particularly in high-income countries, that a framework for systematically measuring and tracking disparities is essential to achieving this aim [2, 3].

This article focuses on health disparities monitoring systems developed in the U.S., which is a high-income country that faces formidable challenges to overcoming health and healthcare disparities, both because it lacks a national healthcare insurance framework, and it has higher poverty and income inequality rates than most OECD countries [4]. Nevertheless, it began to address and document the problem of health disparities in the 1980s, so it has a well-developed systematic infrastructure for health disparities measurement and monitoring [2]. This integrative article identifies lessons from the U.S. experience that are of relevance to other countries, both in terms of the processes through which the health disparities monitoring system was developed, and its content.

We will take Israel as a case in point for applying lessons learned from the U.S. experience, because an initiative of Israeli health policy makers provided the impetus for undertaking this endeavor. In the past decade in particular, the Israeli Ministry of Health (MOH) mobilized resources to develop a comprehensive health disparities reduction program [5]. As a part of its initiative, the MOH Reduction of Health Inequalities Section sought to review the measurement and tracking literature from other countries, with both similar and dissimilar healthcare systems (e.g., those with [U.K.] and without [U.S.] national health insurance) [6], in order to inform Israeli efforts.

Due to the breadth of health disparities field, and the specific questions being raised in Israel to strengthen its health disparities program, this article limits its focus to health disparities monitoring. The description and evaluation of programs/interventions and policies to reduce/eliminate health disparities are beyond the scope of this paper. Clearly, monitoring health disparities is not an end in itself. It is, nevertheless, an essential stepping stone on the path to eliminating health disparities/achieving health equity, and is currently a question of interest for Israeli health policymakers charged with the responsibility reducing health disparities.

## Terminology

We will use the term “inequality parameters” for the factors in which differences in health status and outcomes have been found across population subgroups (e.g., race, ethnicity, sex, age, education, income, geographic region, nativity/immigration status, sexual orientation) [7], and which have been selected for monitoring in efforts to reduce/eliminate health disparities. We will use the term health outcome/indicator for health, healthcare and health determinant factors (including social determinants of health) that are monitored for disparities by the inequality parameters.

## Health disparities and healthcare landscape in Israel

We will preface this integrative article with a brief overview of health disparities and disparities monitoring in Israel because this is the setting from which our research questions arise, and to which we seek to apply the lessons that emerge from the U.S. experience. In Israel, academic publications and data that are routinely collected by various governmental bodies (e.g., the Israel Central Bureau of Statistics [CBS], Israel Centers for Disease Control [ICDC], MOH) have documented disparities between population groups for decades [8–12]. Like the U.S., Israel is a country with higher poverty and income inequality rates than most other OECD countries (even after taxes and income transfers) [13], and persistent disparities in health have been documented along the socio-economic gradient [12]. There are ethnic disparities in health outcomes, disease risk factors, and mortality rates in Israel [9, 14, 15]. The indigenous Arab minority population (which makes up approximately 21% of the total population, and has a poverty rate of 52.6%, compared to that of 13.6% among Israeli Jews [16]) exhibits higher age-adjusted chronic morbidity and mortality, and shorter life expectancy, than the majority Jewish population [8, 9, 17–19]. In addition to this ethnic disparity, the Jewish majority is comprised of groups that differ by ethnicity, and/or nativity/immigration status, across which health disparities have been found. Jews of Middle Eastern/North African origins, and more recent immigrants, have been characterized as having a lower socio-economic position and poorer health outcomes than those of European/North American origins and longer residence/nativity in Israel [14, 20–22]. Disparities by religion or religiosity have likewise been found, as these factors are also aligned with socio-economic status (SES) and/or ethnicity. Disparities in health and healthcare access by geographic region have also been documented, with those living in more peripheral regions having poorer access to certain levels of health care [20, 23]. Many of these inequality parameters intersect or overlap, such that low SES ethnic groups are also likely to live in peripheral geographic regions [16].

Israel enacted a national health insurance law in 1995 that entitled all Israeli residents to access to primary, secondary and tertiary healthcare services, and to a comprehensive and continuously updated “basket” of health technologies (e.g., drugs, devices), regardless of ability to pay [24–26]. Healthcare services are delivered by four not-for-profit health funds [HFs], and paid for by progressive health payroll tax premiums and an allotment of resources to the HFs on the basis of an age, sex and residential area-adjusted capitation formula as a proxy for varying healthcare needs [27].

The National Health Insurance Law represented an important step toward reducing healthcare access disparities, since prior to its enactment, the proportion of uninsured was higher among low SES and minority populations [24, 26, 28]. However, a number of access barriers remain. Shortly after its establishment, the National Health Insurance Law allowed the HFs to begin offering supplemental insurance to cover services not included in the basket of health services, for an additional fee [24]. The subsequent growth of the supplemental insurance programs, together with the introduction (and increase over time) of co-payments for medications, physician visits, medical imaging, etc., has resulted in a clear socio-economic gradient in the utilization of services requiring co-payments [25, 29]. In addition, there is a substantial ethnic disparity in the purchase of supplementary insurance, which reaches 87% among the total adult population, but only 54% among Arab adults [25].

In 2010, the Israel MOH made the reduction of health disparities an official policy priority. It focused primarily on impacting midstream factors (e.g., with in the healthcare system, which are under its direct purview), such as improving access to critical healthcare-service infrastructures in peripheral areas; eliminating financial and other access barriers to care for low-SES population groups; reducing disparities in access to and quality of healthcare services due to cultural barriers; developing incentives and tools that support the efforts of ‘agents of change’ in combating health disparities among target groups, and establishing a national health disparities database [5, 30].

Reporting on and monitoring of health disparities was an integral component of this new policy. Although, as mentioned above, various Israeli governmental and healthcare agencies routinely collect a wealth of data relevant to health disparities [31], there was no consistent methodology or comprehensive database to enable the systematic monitoring of health disparities according to uniform standards in Israel [5]. The MOH health disparities program began producing annual health disparities reports that presented health outcome data from various sources by available inequality parameters [6, 23, 32–35]. A summary of the quantitative data contained in the reports is presented in Additional file 1: Table S1. Disparities monitoring capacities in Israel have been limited by the fact that data on many factors were not consistently collected annually (e.g., Israel CBS Health Survey conducted only in 2009, and Social Survey with battery of health questions conducted in 2010 and 2017). Most of the indicators tracked annually included health outcomes (e.g., infant mortality, life expectancy,) and regional disparities in the distribution of infrastructure/services, hospital beds, and human resources (see Additional file 1: Table S1) [6, 23, 32–35]. Notably, as many of the inequality parameters intersect, the MOH disparities reports sometimes present the health outcome data stratified by more than one inequality parameter simultaneously. This illuminates the compound effect of intersecting inequality parameters (e.g., highest infant mortality rates are found among ethnic minority populations in resource-poor/peripheral regions).

In addition to the MOH health disparity reports, a health disparities knowledge center was established that publishes additional data on health disparities based on in-depth data analyses of the CBS surveys as well as original research [30, 36]. Other organizations, such as the ICDC and the National Program for Quality Indicators in Community Healthcare (QICH) produce periodic reports from survey, surveillance or registry data (ICDC) or aggregate patient data from the HFs (QICH) under their purview [9, 10]. However, information on population characteristics is limited in these data sets, hampering their ability to track health disparities in a comprehensive manner [37].

The important upstream role that social determinants (e.g., education, employment, social services) play in health disparities was acknowledged and discussed (primarily qualitatively) in the MOH health disparities reports, along with updates on indicators of SES inequalities in Israel (e.g., poverty rate, Gini index) [6, 23, 32–35]. However, because of its limited control of policy outside of the healthcare scope, MOH monitoring of and involvement with these upstream factors have been intentionally limited. The MOH health disparities program leaders noted that “finding a way to achieve a substantial impact of each governmental decision on social gaps in general and health disparities in particular …is still a challenge in the Israeli system.” [5, p. 24] Nevertheless, in the past 2 years MOH has committed to a new strategic plan to address health inequalities which includes action items on the social determinants of health such as poverty, racism and social exclusion [38].

## Lessons from the U.S. experience

### Health disparities monitoring in the U.S. was an outgrowth of, and then became an integral part of, population health monitoring

Health disparities monitoring in the U.S. was preceded by initiatives to monitor population health in general that, as a by-product, provided piecemeal evidence of differences in health status and outcomes between racial/ethnic population subgroups [39, 40]. Deliberate, systematic health disparities documentation and monitoring are broadly recognized as being initiated by the Department of Health and Human Services (DHHS) Heckler Report in 1985, which was commissioned in response to the evidence of disparities that had been found through general population surveillance [40].

The Heckler report was instrumental in putting the reduction of health disparities on the national agenda, as an integral component of population health. This was operationalized by explicitly incorporating the reduction of health disparities as one of the goals for improving population health in national programs, such as the Healthy People programs. As Table 1 indicates, the overarching Healthy People goals published from 1990 onward (for the target years 2000, 2010, 2020) explicitly included reducing/eliminating health disparities [41–43]. The scope of this aim was expanded further in the program for the 2020 target year, to include achieving health equity and creating social and physical environments that promote good health [43].

**Table 1:** Health disparities in the Healthy People programs for population health surveillance

Publication year	*1979*	*1990*	*2000*	*2010*
Target year	1990	2000	2010	2020
Overarching Goals*	1) improve infant health and reduce infant mortality 2) improve child health and development and reduce child mortality 3) improve adolescent/young adult health and development and reduce mortality 4) improve adult health and reduce mortality 5) improve older adults’ health and reduce mortality	1) increase the span of healthy life ***2) reduce disparities*** 3) achieve access to preventive services	1) increase quality and years of healthy life ***2) eliminate health disparities***	1) attain high-quality, longer lives free of preventable disease, disability, injury, and premature death ***2) achieve health equity, eliminate disparities, and improve the health of all groups*** 3) create social and physical environments that promote good health for all 4) promote quality of life, healthy development, and healthy behaviors across all life stages
Inequality parameters	[Not defined. Where data allowed: White Black American Indian Hispanic Non-White]	American Indian/Alaska Native Asian/Pacific Islander Black Hispanic Women Adolescents/young adults Older adults People with disabilities People with low SES	Race and Ethnicity American Indian/Alaska Native Asian/Pacific Islander Hispanic/Latino Black (non-Hispanic) White (non-Hispanic) 2 or more races Sex Educational level Income Geographic location Disability status Sexual orientation (data unavailable for all 2010 outcomes)	Race and Ethnicity American Indian/Alaska Native Asian/Pacific Islander Hispanic/Latino Black (non-Hispanic) White (non-Hispanic) 2 or more races Gender Educational level Income Geographic location (rural/urban) Disability status Sexual identity/orientation
Number of Priority Topic Areas	15	22	28	42
Number of Specific Outcomes	226	319	969	1200 (approximately)

*The goals that refer directly to health disparities are emphasized in bold, italic font

The integration of health disparities monitoring into population health surveillance programs such as Healthy People provided it with both a mandate and a formal framework, and made it a cohesive component of population health [41–48]. It also became an integral component of the work of Centers for Disease Control and Prevention (CDC), which carries out a large portion of the national health surveillance activities, and began producing dedicated health disparities reports in 2011 [49]. Furthermore, the Agency for Healthcare Research and Quality (AHRQ) was established with the mandate of monitoring disparities in health care service provision [50, 51]. Health disparities monitoring in the U.S. evolved as these national population health surveillance programs evolved, and its integration into these systems shaped both the selection of the health outcomes/indicators and the inequality parameters that were monitored.

#### Health outcomes/indicators

In the Healthy People programs, all metrics that were identified as important to population health over the past 30 years were also targeted for tracking of health disparities. These outcomes fall into a number of broad categories, including: life expectancy/mortality, morbidity, risk factors, health care services access/quality, and social/environmental determinants of health.

Table 2 lists the priority areas (each of which includes many specific outcomes/objectives) that were tracked in each generation of the Healthy People program, and provides an overview of how this evolved over time.

**Table 2:** Priority Areas of the Healthy People programs 1990-2020

1990 Priority Areas	2000 Priority Areas	2010 Focus Areas	2020 Topic Areas
(*n* = 15)	(*n* = 22)	(*n* = 28)	(*n* = 42)
	*[Clinical preventive services]*	Access to quality health services	Access to quality health services
			Adolescent health
		Arthritis, osteoporosis & chronic back conditions	Arthritis, osteoporosis & chronic back conditions
			Blood disorders & blood safety
	Cancer	Cancer	Cancer
		Chronic kidney disease	Chronic kidney disease
			Dementias
	Diabetes & chronic disabling conditions	Diabetes	Diabetes
	*[with Diabetes]*	Disability & secondary conditions	Disability & health
			Early & middle childhood
	Education & community-based programs	Educational & community-based programs	Educational & community-based programs
Environmental health *[Toxic agent control]*	Environmental health	Environmental health	Environmental health
Family planning	Family planning	Family planning	Family planning
	Food & drug safety	Food safety	Food safety
			Genomics
			Global health
		Health communication	Health communication & health information technology
			Health-related QoL & well-being
			Healthcare-associated infections
		*[with Vision]*	Hearing & other sensory or communication disorders
High blood pressure control	Heart disease & stroke	Heart disease & stroke	Heart disease & stroke
	HIV infection	HIV	HIV
Immunizations Infectious agent control	Immunizations & infectious diseases	Immunizations & infectious diseases	Immunizations & infectious diseases
Injury control *[accidental]*	Injuries *[unintentional]* Violent and abusive behavior	Injury & violence prevention	Injury & violence prevention
			Lesbian, gay bisexual & transgender health
Maternal *[Pregnancy]* & infant care	Maternal & infant health	Maternal, infant & child health	Maternal, infant & child health
		Medical product safety	Medical product safety
	Mental health & mental disorders	Mental health & mental disorders	Mental health & mental disorders
Nutrition	Nutrition	Nutrition & overweight	Nutrition & weight status
Occupational safety & health	Occupational safety and health	Occupational safety & health	Occupational safety & health
			Older adults
Fluoridation of water supplies	Oral Health	Oral health	Oral health
Physical activity *[Exercise & fitness]*	Physical activity/fitness	Physical activity/fitness	Physical activity
			Preparedness
	*[*Clinical preventive services*]* *[Surveillance & data systems]*	Public health infrastructure	Public health infrastructure
		Respiratory diseases	Respiratory diseases
Sexually transmissible diseases	Sexually transmitted diseases	Sexually transmitted diseases	Sexually transmitted diseases
			Sleep health
			Social determinants
Stress management			
Substance use *[Alcohol & drugs]*	Substance abuse	Substance abuse	Substance abuse
Tobacco *[smoking cessation]*	Tobacco use	Tobacco use	Tobacco use

The Healthy People 1990 program identified 15 priority areas (Table 1) and set 226 measurable health objectives to be used to track population health [44]. Since reducing health disparities was not an a priori aim of this program, only a small number of these objectives could be used to explore differences between population groups, with a very limited subset of inequality parameters [45].

The Healthy People 2000 initiative, which set reducing health disparities as an a priori aim, identified 22 priority areas to be tracked (Table 1) and 319 national objectives to be achieved. All priority areas and national objectives were evaluated by the health disparities parameters, wherever data permitted. Systematic documentation and reporting of gaps in the data (by the inequality parameter subcategories) also began in this period [44].

The commitment to solving the problem of health disparities continued to grow, and both Healthy People 2010 and 2020 set *eliminating* health disparities as a main goal [46, 47]. Healthy People 2010 identified 28 priority areas (Table 1), and set over 900 specific health promotion and disease prevention objectives to track progress [44]. Additional priority areas introduced in the 2010 program included outcomes related to access to and quality of care, a broader range of specific chronic conditions, and technological advances (e.g., health communication, medical devices; Table 2) [46].

Healthy People 2020 identified 42 priority areas, adding areas focused on life stages, health-related quality of life, genomics, global health, and social determinants [46, 47].

While the Healthy People programs have evolved to track a rather overwhelming number of indicators (~ 1200), they also identified a much smaller subset of “Leading Health Indicators” (LHI) that reflect the major public health concerns in the U.S. These LHI were selected on the basis of: 1) their ability to motivate action, 2) the availability of data to measure their progress, and 3) their relevance as broad public health issues [46]. Additional file 2: Table S2 lists the 12 LHIs of the Healthy People 2020 program with the target for each indicator. The table presents information extracted from the data page of each LHI on the Healthy People 2020 website [48], including the target to be reached by 2020, and baseline and most recent data both for the population as a whole and for selected inequality parameters.

The CDC, which hosts the National Health Center for Health Statistics, is responsible for collecting much of the nationally representative data that it and other agencies/initiatives use to monitor public health and health disparities. From the plethora of data collected under its auspices, the CDC set the following criteria for selecting health indicators/topics to include in their health disparities reports:

1. The data must be of high quality and appropriate for developing national estimates.
2. In addition, the topic had to meet one or more of the following criteria:
  - leading cause of premature death, higher disease burden, or lower life expectancy at birth for certain segments of the U.S. population as defined by sex, race/ethnicity, income or education, geography, sexual orientation, and disability status;
  - known determinant of health (e.g., social, demographic, and environmental) where disparities have been identified; and/or
  - health outcome for which effective and feasible interventions exist where disparities have been identified [49].

The AHRQ is a DHSS agency that generates measures and data on the quality of healthcare in the U.S. As a part of national efforts to reduce health disparities, in 1999 the AHRQ began producing an annual National Healthcare Disparities report [50]. Its reports focus on: a) measures of access to and quality of care; and b) the National Quality Strategy (NQS) priorities, which include: patient safety, person-centered care, care coordination, effective treatment, healthy living, and care affordability [51].

#### Inequality parameters

There was initially very little discussion in the U.S. literature about the ‘selection’ of inequality parameters to track in health disparities monitoring. It rather seems that the inequality parameters were self-evident, or self-selected, based upon available evidence of differences in health outcomes for, and/or evidence of discrimination against, specific population groups [52].

However, the various health disparities monitoring programs discovered that the lack of consistent, widely used standards for collecting and reporting health data by racial, ethnic and other inequality parameters complicated the documentation of health disparities [2, 53, 54]. As a consequence, in 2011 the DHHS set minimum data standards for race, ethnicity, sex, primary language, and disability status to be implemented in all federally funded population health surveys where person-level data were collected. The race/ethnic-origin data standards included a more granular list of 18 categories, breaking down the Asian and Hispanic groups to geographic-origin distinct subcategories whenever feasible. In addition, more detailed information was collected on primary language and language proficiency, and on physical disabilities and limitations [55]. It is noteworthy, however, that the 2011 Standards did not include any SES parameters in the standards, nor was this even discussed in the documentation explaining the standards [55].

Additional file 3: Table S3 presents a summary of the 3 major health disparities monitoring and reporting initiatives described above (Healthy People, CDC, AHRQ). The inequality parameters surveilled for health disparities are quite similar for the CDC and the Healthy People 2020 initiatives and reflect the 2011 DHHS standards [55]. For some of the population domains (e.g., sexual orientation, primary language) data are still largely unavailable (indicated in gray rather than black font in the table) [47, 49].

The CDC and Healthy People 2020 also track many of the same health outcomes/indicators. However, Healthy People 2020 tracks utilization of healthcare services, specific disease trajectories, psycho-social indicators, and some SES, environmental and lifestyle indicators in more detail than the CDC does [56]. While the role of the CDC is primarily monitoring and reporting, the Healthy People initiative relates the data to targets (shown in the Healthy People 2020 column of Table 2 in parentheses, where targets have been set), and reports on population progress toward meeting the targets.

The AHRQ National Healthcare Quality and Disparities Reports include more than 250 measures of quality and disparities covering a broad array of health care services and settings [57–59]. In more recent years, detailed disparity information is also available in supplementary (chart book) reports for each of the NQS priorities [60–63].

### High-level political commitment and legislation played an important role

High-level political support and federal/national legislation have played an important role in establishing systems for health disparities monitoring in the U.S. The Heckler Report was commissioned by the DHHS Secretary, and given this top-level political commitment to the problem of health disparities, the DHHS established an Office of Minority Health in 1986. This office was subsequently authorized and reauthorized in legislation passed in 1990, 1998, and 2010 [64]. Additional congressional legislation in 1999 required the AHRQ to produce annual National Health Care Disparities Reports [65]. Congress also passed the Minority Health and Health Disparities Research and Education Act of 2000. This act commissioned the Institute of Medicine (IOM, an independent, non-governmental organization of eminent professionals that guides national health policy) to conduct a comprehensive study of the DHHS health disparities data collection systems. It also required the National Institutes of Health (NIH) to establish the National Center on Minority Health and Health Disparities (NCMHD) [52]. Within its charge of developing and implementing NIH-wide strategic plan for health disparities research, the NCMHD was given responsibility for supporting research that identified the most critical health disparities factors/outcomes to monitor, and the best ways to measure them.

During another period of commitment to eliminating health disparities in the U.S. at the very highest political level, the 2010 Affordable Care Act (ACA) was passed. It mandated the establishment of Minority Health offices in 6 other DHHS agencies, including AHRQ and CDC [66]. In addition, it elevated the status of the NCMHD to an NIH institute (National Institute on Minority Health and Health Disparities [NIMHD]) with responsibility for further refining and developing definitional and methodological issues in health disparities research, and coordinating cross-institute and interdepartmental health disparities research. The NIMHD launched a Resource-Related Health and Health Disparities Research Initiative, and established the Data Infrastructure and Information Dissemination on Health Disparities Research Initiative. It also established a National Health Disparities Research Coordinating Center (NHDRCC) to collect, integrate and track data on health disparities research. In addition, the NHDRCC was charged with analyzing and interpreting data from a variety of research projects to facilitate reporting on progress and gaps in health disparities research, and approaches to understanding health disparities. It provides a central source of links to racial and ethnic health and health care disparities reports [67], several of which provide excellent models for health disparities data surveillance and reporting [60–63, 68].

Clearly, health disparities monitoring programs can be advanced by governing powers with a political philosophy or ideology that promotes equality, fairness and the rights of minority populations; and, in equal measure, can be undermined by governing powers that are indifferent or even hostile to these principles. This is illustrated by recent events in the U.S. While the ACA reforms explicitly provided for health disparities reporting and research to track health disparities, the recently proposed ACA replacement acts of the current administration did not mention health disparities, nor have any provisions calling for monitoring them [69, 70].

Since initial governmental commitments were made to tracking and reducing health disparities in the 1980s, there have been a number of American administrations with varied visions for public health. It seems, however, that the systematic monitoring of health disparities has remained on track, perhaps because it has been incorporated into 10-year programs for total population health surveillance, or mandated by legislation that is not easily repealed, despite changes in the priorities of successive administrations. Nevertheless, the current political period is likely to shed light on how robust the U.S. program for monitoring and eliminating health disparities is in the absence of administration-level support, and on what other sources of support may emerge.

### The monitoring of health outcomes/indicators and inequality parameters provided a feedback loop that informed future changes in/expansion of the outcomes/indicators and parameters monitored

This section examines in more depth how the health disparities monitoring process led to an evolution in the fundamental understanding of the causes of health disparities, which in turn let to changes in the outcomes and inequality parameters that were monitored.

This is particularly evident in the Healthy People program. As 2010 approached, evaluations of progress toward meeting the health outcome targets led to a shift in the understanding of health disparities, as well as in the outcomes selected for monitoring for the coming decade. The IOM issued a report which showed that progress toward targets occurred for about half of the leading health indicators; however, there was no significant change in disparities for about 70% of the leading health indicator objectives [71]. The IOM report raised issues that had not thus far been monitored, such as the negative effects of racism, residential segregation, and low SES [71]. It was joined by other researchers in recognizing that “macro-level factors and systemic forces are what fundamentally drive population level inequities. Research and interventions, therefore, should target these factors operating at the macro levels of the socioecologic framework.” [72, p. 1395].

The work of additional non-governmental organizations (e.g., Robert Wood Johnson Foundation, Kellogg Foundation, the California Endowment, Kaiser Family Foundation, MacArthur Research Network on Socioeconomic Status and Health) on health disparities raised similar concerns [71]. One of their critiques was that the early governmental initiatives to address health disparities tended to focus primarily on individual-level risk factors and medical care interventions. They acknowledged that reducing disparities in medical care was essential, but also compiled data showing that the effective prevention/management of many health problems did not lie principally in hospitals and doctors’ offices, but rather in the broader environment (e.g., homes, schools, workplaces, playgrounds and parks, grocery stores, sidewalks and streets, air, water) [73]. As a result, they aimed to expand the view of what it means to be healthy from looking only at where health ends (e.g., disease and healthcare system outcomes), to looking also at where health begins (e.g., social, economic and physical living conditions) [74], and developing policies and programs that would break down barriers to good health, particularly for those who faced the greatest obstacles [75].

In light of these initiatives, Healthy People 2020 adopted a framework that viewed individual-level and population-level factors as complementary elements of an integrated, comprehensive strategy for disease prevention and health promotion [76]. Its principal, and indeed, primary focus was centered on the social determinants of health as the “root causes of health disparities” [43, p. 29]; while health care was considered “a secondary focus” [43, p. 20].

The Healthy People 2020 website introduced “Social Determinants” as a new priority area with outcomes related to the social aspects of these upstream determinants (e.g., access to educational, economic, job, transportation and affordable housing opportunities; quality of education/job training; food security; public safety/exposure to crime, violence and social disorder; concentrated poverty; residential segregation; incarceration; political participation); as well as the physical aspects (e.g., natural environment/green spaces; built environment; housing and community design; exposure to toxic substances) [77, 78]. The new life stage priority areas (e.g., Early and Middle Childhood, Adolescent Health, Older Adults) tracked additional social determinants; including, for example, intermediate educational access and achievement, and access to social services in each life stage as outcomes. The CDC adopted a similar emphasis on social, economic, and environmental factors as some of the strongest predictors of health in its 2013 report on health disparities, and defined social/environmental determinants as outcomes in health disparities monitoring [49]. These ‘social determinant-outcomes’ were monitored by the inequality parameters, because just as with the more traditional health and risk factor outcomes, differences in the social determinant-outcomes across sex, race/ethnicity, region and sexual orientation categories represented unnecessary and unjust differences in health opportunities/potential, which would subsequently translate into unnecessary and unjust differences in health.

As some of these social determinants of health (e.g., the educational and income/poverty variables) were traditionally used as inequality parameters, and continue to be used as such, their classification as outcomes represents a paradigm change of substantial significance. The use of educational achievement as an inequality parameter, for example, implies the need to eliminate health differences across differing levels of educational achievement, while differences in educational achievement are taken as a given. In contrast, the use of educational achievement as a social determinant-outcome implies that the differences in educational achievement must be monitored and eliminated in order to eliminate health disparities. This also implies that the interventions needed to address health disparities cannot be confined to the healthcare system, but must target social, economic and physical conditions critical to health. The latter approach is consistent with the understanding of and emphasis on the social determinants of health articulated in the Healthy People 2020 and recent CDC health disparities programs. However, the rationale for using the same metric (e.g., educational achievement) both as an inequality parameter and as an outcome was not addressed in the program documentation [49, 76, 77]. This introduces a source of confusion; and the need for a differentiated, more precise nomenclature. Explicitly addressing this dilemma would lead to better refining and directing health disparities program policy and elimination efforts. For example, educational achievement should perhaps not be used as an inequality parameter, with its inference that differences within this parameter are unmodifiable/not of concern. It should rather only be used as a social determinants-outcome, given that reducing disparities in educational achievement is a precondition to reducing health disparities.

The inequality parameters used to monitor health disparities have also evolved over time, as can be seen across the generations of the Healthy People program (Table 1), due at least in part to feedback from expanded and more systematic health disparities monitoring and better data availability [41–46, 79]. In Healthy People 2000, the list of “special populations” to be tracked for disparities included: the major racial/ethnic minority groups, women, adolescents/young adults/older adults, and low SES categories (Table 1) [41]. In the Healthy People 2010 program the category of ‘people with low SES’ was replaced with the categories of educational level and income. In addition, the categories of geographical location (rural/urban) and sexual orientation were added [44].

Healthy People 2020 began tracking additional inequality parameters as a natural outgrowth of its focus on the social determinants of health. More extensive use was made of data available from a broad range of governmental departments to introduce parameters of inequality that were further upstream than the traditional inequality parameters.

For example, for the Healthy People 2020 outcome of the percentage of 4th graders who are at or above grade level for reading skills, in addition to the standard inequality parameters, data were presented by educational attainment of the parents, school type (public/charter/private), school lunch program eligibility, native English speaker status, and type of community where school is located [80]. The use of these additional inequality parameters/sub-parameters provides vital information about how intergenerational and community disparities impact intermediate educational attainment, on the path to adult educational attainment; which in turn, determines health and mortality outcomes and disparities throughout the life course.

A number of social scientists and epidemiologists have critiqued that fact that the collection of and reporting on inequality parameters in the U.S. (e.g., race/ethnicity, class, gender) presents them as independent and individualized traits; although, in reality, the core inequality parameters often cluster together [52, 81–83]. As such, health disparities data need to be collected and presented in a format that allows for using analytical techniques that explore the intersectionality of the inequality parameters (e.g., joint health consequences of being a low-SES, racial/ethnic minority female) and its effects on health trajectories over the life course/intergenerationally (e.g., multiple-hierarchy stratification) [83]. Such an approach can begin to elucidate the social relations of power that determine the clustering of disadvantage, and that need to be addressed in order to eliminate health disparities [82].

### Data monitoring has served as a tool to identify data gaps and provided an impetus for developing plans to close the gaps

As the various health disparities monitoring initiatives determined inequality parameters and health outcomes of interest and began tracking them, they discovered that data were unavailable for many inequality parameters. Nevertheless, the gaps in data were themselves systematically documented and used to improve the health disparities monitoring system. In the final Healthy People 2000 report, these data issues were explicitly addressed and specific objectives were set, calling for: 1) identifying gaps in the data; and 2) establishing mechanisms to meet data needs for more granular racial/ethnic subgroups (e.g., American Indian/Alaska Native, Asian/Pacific Islander, Black, Hispanic/Latino), and low SES categories [44].

The Healthy People 2010 documents also very clearly addressed remaining gaps in the data, and called for multiple actors at different levels of the system and society to work to fill those gaps. They noted, for example, that data by sexual orientation were unavailable for all Healthy People 2010 outcomes.

In addition, in the 2010 final report, the documentation and reporting of gaps in data by health disparities parameters by each specific objective/outcome became much more systematic and explicit [46]. Health Disparities Tables were created for the Priority Topic Areas and the Leading Health Indicators, which summarized the data availability and status of each specific objective/outcome by the inequality parameters. Additional file 4: Table S4 displays the Health Disparities Table for Leading Health Indicators from the final report of Healthy People 2010 [46], and provides a salient example of how a very large volume of disparities data can be effectively summarized and presented, while visually highlighting data gaps. According to a color-coded legend, the best group rate within each inequality parameter is identified. In addition, the extent of the disparity from the best rate among the other groups within the inequality parameter is indicated, as well as whether the magnitude of the disparity is increasing or decreasing. If data is unavailable by any inequality parameter (or for any sub-group within an inequality parameter), this is also explicitly indicated in the table (see legend at end of Additional file 4: Table S4). For example, a review of the objective “19-2.Obesity in adults” by the Race/Ethnicity parameter in Additional file 4: Table S4 shows that non-Hispanic Whites had the best (lowest) rate; Blacks and Hispanics differed from the best rate by 10-49%; the disparity between Hispanics and the best group rate decreased since 2000; and there were no data for four other racial/ethnic groups.

Summary reports of the Healthy People 2010 program indicated that 40% of the objectives could not be assessed, particularly as related to health disparities [43]. Setting developmental objectives, despite the lack of baseline or tracking data, was identified as an important first step to stimulating the creation of data collection systems [43].

Data availability continues to be monitored and reported in the web presentation of the data for Healthy People 2020. For example, the adult obesity outcome data table includes sexual orientation and gender identity, with the notation that data is unavailable ([84], see the “View data by group” tab). The Healthy People 2020 also program put a priority on developing data collection objectives for any outcomes/objectives critical to achieving health equity, for which data were lacking [76].

### Health disparities monitoring in the U.S. has become a multi-agency, cross-departmental effort

Health disparities monitoring has grown into a multi-agency endeavor, within and beyond the DHSS. The DHSS Minority Health agencies established by the ACA formed a DHSS Health Disparities Council which developed and oversees broad health disparities plans/activities that affect the way nationwide health data is collected. Its 2011 plan called for implementing a multifaceted health disparities data collection strategy across the DHHS, which aimed to:

- Establish data standards and ensure federally conducted or supported health care or public health programs, activities, or surveys collect and report data in five specific demographic categories: race, ethnicity, gender, primary language, and disability status as authorized in the Affordable Care Act;
- Oversample minority populations in DHHS surveys;
- Develop other methods for capturing low-density populations (e.g., Native Americans, Asian Americans and Pacific Islanders), when oversampling is not fiscally feasible;
- Use analytical strategies and techniques, such as pooling data across several years, to develop estimates for racial and ethnic minority populations;
- Publish estimates of health outcomes for racial and ethnic minority populations and subpopulations on a regular, pre-determined schedule;
- Make aggregately-collected healthcare service quality measurement data that call attention to racial and ethnic disparities publicly available;
- Improve public access to DHHS minority data and promotion of external analyses; and
- Develop and implement a plan for targeted special population studies, internally or through research grant funding announcements and contracts. This initiative will also address gaps in subpopulations traditionally missed by standard DHHS data collection activities [54].

Expanding efforts both within and beyond the DHHS, recent U.S. government initiatives were directed at creating a broad, comprehensive, and coordinated national approach. The DHHS approach of promoting “health in all policies” entailed working cross-governmentally and engaging agencies such as the U.S. Departments of Justice, Education, Labor, Transportation, etc. to more directly and effectively address the social determinants of health [76].

The Affordable Care Act created the inter-departmental (e.g., inter-ministry) National Prevention, Health Promotion and Public Health Council (NPC). The elimination of health disparities was one of 4 strategic directions in its National Strategy, and included supporting research to identify effective strategies to eliminate health disparities, and standardizing and collecting data to better identify and address disparities [85]. The vast breadth of the federal/national governmental departments, agencies and offices included in the NPC (see Box 1) provides an important model of the expansion required in order to monitor disparities across the full range of social and environmental determinants of health. The NPC strategy also proposed partnerships with state, tribal, local and territorial governments; businesses and employers; healthcare systems, insurers and clinicians; early learning centers, schools, colleges, and universities; community, non-profit, and faith-based organizations; and individuals and families [85].

**Box 1 Members of National Prevention, Health Promotion and Public Health Council (NPC)**

**Table:** 

• Bureau of Indian Affairs
• Corporation for National and Community Service
• Department of Homeland Security
• Department of Defense
• Department of Justice
• Department of Labor
• Department of Transportation
• Domestic Policy Council
• Department of Education
• Environmental Protection Agency
• Federal Trade Commission
• Department of Health and Human Services
• Department of Housing and Urban Development
• Office of Management and Budget
• Office of National Drug Control Policy
• Department of Veterans Affairs

The NPC Strategy actions specifically related to disparities monitoring included:

- identifying and mapping high-need areas that experience health disparities and aligning existing resources to meet these needs, and
- increasing the availability of de-identified national health data to better address the needs of underrepresented population groups [86].

### U.S. health disparities programs are exploiting the potential of digital tools to improve the reach and timeliness of disparities monitoring and reporting

The DHHS and other governmental agencies make a wealth of health disparities monitoring data and reports (both historic and current) freely available on the web. Most recently, the use of a web platform for the Healthy People 2020 program and data tracking makes the data very accessible to public health researchers, policy makers, and the general public. This facilitates moving beyond simply collecting data to making the data available to address health disparities in a more timely and continuous manner. The Healthy People 2020 web platform brings together a great wealth of data from other governmental agencies (e.g., Departments of Education, Labor, Justice, Housing and Urban Development, etc.) that are systematically included in health disparities outcome reporting, by all available inequality parameters.

The potential of electronic health records (EHRs) has also been recognized in the U.S. as a rich source of yet untapped data that could be very useful in addressing health disparities. The incorporation of a screening tool for social determinants of health into the EHR could give providers and health care systems, policymakers, and public health practitioners a granular sense of issues related to the health disparities across racial/ethnic and other inequality parameters. Setting metrics for relevant outcomes could drive the process of improving the value of health systems data for disparities monitoring [87].

## Application in the Israeli context

U.S. health disparities monitoring programs provide useful lessons for the international community, and we will now consider how these lessons might be applied to the Israeli context. The starting point for this article was the interest of the Israeli MOH in choosing a set of health outcomes/indicators/determinants and inequality parameters to be used for the systematic monitoring of health disparities in Israel. Resources such as the Health People Leading Indicators, and the CDC health disparities reports provide useful and specific criteria for choosing outcomes. In addition, the U.S. experience of explicitly defining inequality parameters, and requiring, through legislation, that health outcome data be collected by these parameters in DHHS/government surveys, may also be applicable in Israel.

Use of the monitoring process, as was done in the U.S., to systematically identify gaps in the data for specific inequality parameters/categories, would make it possible for Israeli policymakers to develop plans for closing the data gaps and building a comprehensive health disparities monitoring system.

The Israeli health disparities program has thus far dedicated most of its resources and its most consistent monitoring efforts to tracking and reducing the impact of low SES/regional disparities and language/cultural differences on access to health services. It has, nevertheless, from the outset, recognized the critical role of the social determinants of health. Israeli governmental agencies/ministries collect a wealth of data that could be used for systematic monitoring of disparities in the social determinants of health. The MOH has addressed the need for horizontal and vertical, cross-ministry cooperation to implement such a monitoring system. One of the major challenges that it continues to confront is the difficulty of “leveraging the problem of inequity into a pan-governmental responsibility” [30, p. 10].

Although direct action to affect the social determinant-outcomes is largely beyond the purview of the MOH, the U.S. model of formally tracking them from data routinely collected by other governmental departments is relevant to the Israeli context as well. The MOH health disparities program has quite consistently reported on poverty and income inequality rates. The U.S. model shows how this can be strengthened and enriched by tracking inequalities in their predecessors, such as educational achievement, employment, etc. In addition, existing, publically accessible data makes it possible to track these social determinant-outcomes by relevant sub-population disparities parameters, which is something that was not done in the MOH social determinants of health disparity reporting to date. Such routinized data tracking and reporting would provide the MOH with an evidence base to help it to more effectively achieve its commitment of:

Active involvement … in maintaining awareness at the highest decision-making echelon of the importance of narrowing social gaps, and the high priority that should be given to this struggle. The health authority should emphasize the relationship between social disparities and health disparities and the need for a national endeavor to tackle them. It is recommended that the health authority leader should present the government with an annual update on progress in this arena [5: p. 23].

Finally, the use of information technology in the Israeli healthcare system is extensive, and comprehensive data on healthcare utilization and morbidity is collected in the digital databases of the four HFs and by the MOH. Furthermore, information on the social determinants of health (e.g., education, employment, income) by inequality parameters (e.g., ethnicity, immigration/nativity, geographic region) is regularly collected by various government departments (e.g., Ministry of Education, National Insurance Institute of Israel, Israel Central Bureau of Statistics). The linkage of these national-level data on the social determinants of health to data on health outcomes and health service utilization would provide an unparalleled resource for understanding, tracking, and intervening to eliminate health disparities. However, these datasets have not been systematically linked in practice due to legal, organizational, financial and other barriers preventing data sharing and secondary use of healthcare data in Israel. This issue was recently discussed in a workshop for national-level health policy executives, and the workshop summary called for regulatory action to reduce these barriers [88].

## Conclusion

The U.S. infrastructure for health disparities monitoring provides a model of global relevance in terms of how inequality parameters and outcomes can be determined, how data collection systems can be established (using legislative measures and broad cross-governmental resources), and how data gaps can be identified, tracked and eliminated. It also provides a model for how disparities data can be shared to support the design and implementation of policy, clinical and community interventions. Furthermore, it highlights the value of high-level political commitment and legislation.

To effectively translate these health disparities measurement and tracking lessons into practice in Israel, a number of changes are needed. First, there is a need to better incorporate social determinants of health as health disparity *outcomes/indicators* that are tracked and analyzed according to inequality parameters. Second, adequate political and budgetary resources must be allocated to support: 1) systematic data collection that meets the needs of health disparities research, 2) the synthesis and linkage of existing cross-departmental/ministerial data for the purposes of tracking and eliminating health disparities, and 3) making health disparities data (in addition to summary analyses) as accessible and widely available as possible, by the broadest range of inequality parameters and disparity outcome targets. Finally, legal barriers (whether real or artificial) to access to and the linkage of de-identified data from various levels of the healthcare system and other relevant governmental datasets must be removed.

## Additional files

Additional file 1: **Table S1.** Health outcome data presented in the MOH Health Disparities reports by inequality parameter and report year. (PDF 476 kb)

Additional file 2: **Table S2.** Healthy People 2020 Leading Health Indicators at baseline and most recent year for selected inequality parameters. (PDF 419 kb)

Additional file 3: **Table S3.** Summary of inequality parameters and health indicators in key health disparities monitoring initiatives in the U.S. (PDF 446 kb)

Additional file 4: **Table S4.** Healthy People 2010 Leading Health Indicators by inequality parameters. (PDF 1379 kb)

## Abbreviations

ACA: Affordable Care Act
AHRQ: Agency for Healthcare Research and Quality
CBS: Central Bureau of Statistics (Israel)
CDC: Centers for Disease Control and Prevention
DHHS: Department of Health and Human Services
EHR: Electronic health record
FIHET: Federal Interagency Health Equity Team
HF: Health Fund
ICDC: Israel Centers for Disease Control
IOM: Institute of Medicine
NHDRCC: National Health Disparities Research Coordinating Center
NHLBI: National Heart, Lung, and Blood Institute
NIMHD: National Institute on Minority Health and Health Disparities
NPA: National Partnership for Action
NPC: National Prevention, Health Promotion and Public Health Council
NQS: National Quality Strategy
OECD: Organization for Economic Cooperation and Development
QICH: Quality Indicators in Community Healthcare (Israel National Program)
REACH: Racial and Ethnic Approaches to Community Health
SES: Socioeconomic status
