# Supplementary material for: Health disparities monitoring in the U.S.: lessons for monitoring efforts in Israel and other countries
Source: Isr J Health Policy Res. 2018 Feb 28;7:14. doi: 10.1186/s13584-018-0208-1 (PMC6389259; doi:10.1186/s13584-018-0208-1)
Supplement: Supplementary file 1 — Table S1. Health outcome data presented in the MOH Health Disparities reports by inequality parameter and report year. (PDF 476 kb) [file 13584_2018_208_MOESM1_ESM.pdf]

Supplemental Table 1. Health outcome data* presented in the MOH Health Disparities reports by inequality parameter and report year

Outcome/ Indicator	Ethnicity						SES†						Sex						Age						Region						Immigration/ nativity						Marital status						Religiosity						Mother tongue					
	2010	2012	2013	2014	2015	2016	2010	2012	2013	2014	2015	2016	2010	2012	2013	2014	2015	2016	2010	2012	2013	2014	2015	2016	2010	2012	2013	2014	2015	2016	2010	2012	2013	2014	2015	2016	2010	2012	2013	2014	2015	2016	2010	2012	2013	2014	2015	2016	2010	2012	2013	2014	2015	2016
**Morbidity/mortality**																																																						
Life expectancy																																																						
Infant mortality (e.g., all-cause, cause-specific)																																																						
Mortality (e.g., all-cause, cause-specific)																																																						
Suicide																																																						
Morbidity (e.g., CVD, HTN, DM)																																																						
Morbidity, asthma																																																						
Morbidity, cancers																																																						
Self-reported health problem																																																						
Self-rated health																																																						
Self-rated/reported mental health																																																						
Emotional well-being																																																						
Physical disability																																																						
Activities of daily life																																																						
**Health behaviors**																																																						
Physical activity																																																						
Physical activity among those with chronic disease																																																						
Smoking																																																						
Smoking among those with chronic disease																																																						
Overweight/obesity																																																						

Outcome/ Indicator	Ethnicity						SES†						Sex						Age						Region						Immigration/ nativity						Marital status						Religiosity						Mother tongue					
	2010	2012	2013	2014	2015	2016	2010	2012	2013	2014	2015	2016	2010	2012	2013	2014	2015	2016	2010	2012	2013	2014	2015	2016	2010	2012	2013	2014	2015	2016	2010	2012	2013	2014	2015	2016	2010	2012	2013	2014	2015	2016	2010	2012	2013	2014	2015	2016	2010	2012	2013	2014	2015	2016
Alcohol consumption																																																						
Went without food due to lack of money																																																						
**Health Services**																																																						
Doctors/1,000 people																																																						
Nurses/1,000 people																																																						
Dentists/1,000 people																																																						
Para-medical personnel/1,000 people																																																						
Specialists/10,000 people																																																						
Hospital beds/positions (total, for specific departments)/1,000 people																																																					
Hospitalization rates																																																						
Maternal and child health centers																																																						
Pharmacies																																																						
Hemodialysis centers																																																						
Salary gaps among and between health professionals																																																						
Quality indicators in hospitals																																																						
Patient experience of hospitalization																																																						
Waiting time to get appointments																																																						

Outcome/ Indicator	Ethnicity						SES†						Sex						Age						Region						Immigration/ nativity						Marital status						Religiosity						Mother tongue					
	2010	2012	2013	2014	2015	2016	2010	2012	2013	2014	2015	2016	2010	2012	2013	2014	2015	2016	2010	2012	2013	2014	2015	2016	2010	2012	2013	2014	2015	2016	2010	2012	2013	2014	2015	2016	2010	2012	2013	2014	2015	2016	2010	2012	2013	2014	2015	2016	2010	2012	2013	2014	2015	2016
Accessibility of:																																																							
Emergency rooms																																																							
Community emergency services																																																							
Family doctors (community)																																																							
Specialists																																																							
Gave up on medications or medical/dental services																																																							
Self-reported language barrier to healthcare																																																							
Trust in and satisfaction with healthcare system																																																							
Use of private doctors																																																							
Use of dental services																																																							
Household health care expenditures																																																							
**Preventive health**																																																							
Cancer screening tests (mammography, fecal occult blood test)																																																							
Influenza immunization																																																							
Childhood immunizations																																																							
Diabetes control (among diabetics)																																																							

Outcome/ Indicator	Ethnicity						SES[†]						Sex						Age						Region						Immigration/ nativity						Marital status						Religiosity						Mother tongue					
	2010	2012	2013	2014	2015	2016	2010	2012	2013	2014	2015	2016	2010	2012	2013	2014	2015	2016	2010	2012	2013	2014	2015	2016	2010	2012	2013	2014	2015	2016	2010	2012	2013	2014	2015	2016	2010	2012	2013	2014	2015	2016	2010	2012	2013	2014	2015	2016	2010	2012	2013	2014	2015	2016
Blood cholesterol test done (adults)																																																						
**Social/ Environmental determinants**																																																						
Income																																																						
Employment																																																						
Housing density																																																						
Homicide rate																																																						
Voter participation																																																						
Internet access																																																						
Air quality																																																						
Social support																																																						

[*]The data presented in the MOH health disparity reports [5, 22, 32-35] is collected by a number of different agencies, including the MOH, the Central Bureau of Statistics, the Israel Centers for Disease Control, disease-specific registries, Health Funds, etc. Gaps in the health disparities reports are often due to gaps in data availability, as not all agencies collect the data on an annual or on-going basis.

[†]SES-socioeconomic status, is represented by different indicators for different outcomes and across different years, including educational level, income level, labor force participation, property ownership, SES ranking of town, exemption from healthcare service co-payments (due to low SES), geocodes, etc.
