# Supplementary material for: Health disparities monitoring in the U.S.: lessons for monitoring efforts in Israel and other countries
Source: Isr J Health Policy Res. 2018 Feb 28;7:14. doi: 10.1186/s13584-018-0208-1 (PMC6389259; doi:10.1186/s13584-018-0208-1)
Supplement: Supplementary file 2 — Table S2. Healthy People 2020 Leading Health Indicators at baseline and most recent year for selected inequality parameters. (PDF 419 kb) [file 13584_2018_208_MOESM2_ESM.pdf]

Supplemental Table 2. Healthy People 2020 Leading Health Indicators at baseline (BL) and most recent (MR) year for selected inequality parameters

Indicator	Target	Total		White		Black		Hispanic*		< High school		4y college degree	
		BL	MR	BL	MR	BL	MR	BL	MR	BL	MR	BL	MR
**Access to health services**													
% persons (<65y) with medical insurance	***100.0***	83.2	89.4	87.5	92.5	82.1	88.8	66.7	78.9	56.9	68.1	91.6	95.0
% persons with usual primary care provider	***83.9***	75.6	76.5	79.0	79.8	72.1	73.0	77.6	78.1	64.6	68.3	75.2	75.9
**Clinical preventive services**													
% adults receiving colorectal cancer screening per guidelines (age adjusted, 50–75 y)	***70.5***	52.1	62.4	55.1	65.4	48.6	61.3	53.8	64.1	35.4	46.0	57.6	67.4
% adults with hypertension with controlled blood pressure (age-adjusted, %, ≥18y)	***61.2***	43.7	50.3	46.5	54.8	41.2	43.1	31.8	40.9	36.5	50.7	50.2	53.7
% persons with diagnosed diabetes whose A1c value is >9% (age-adjusted, ≥18y)	***16.2***	18.0	20.5	13.1	14.6	27.3	25.5	26.0	26.0	22.5	23.4	--	20.8
% fully immunized young children (19–35 mos.)	***80.0***	68.4	71.6	69.3	72.6	64.8	65.4	67.8	74.3	63.4	68.0	74.7	76.2
**Environmental quality**													
% children exposed to secondhand smoke (nonsmokers, 3–11y)	***47.0***	52.2	41.3	55.1	39.4	67.4	67.9	30.8	29.1	--	--	--	--
**Injury & violence**													
Injury deaths (age-adjusted, per 100,000 population)	***53.7***	59.7	60.1	62.2	66.5	67.0	60.7	61.7	63.7	--	--	--	--
Homicides (age adjusted, per 100,000 population)	***5.5***	6.1	5.1	2.8	2.4	21.9	18.2	6.8	4.5	--	--	--	--
**Maternal, infant & child health**													
Infant deaths (per 1,000 live births, <1y)	***6.0***	6.7	6.0	5.6	5.1	13.4	11.1	5.4	5.0	--	--	--	--

Indicator	Target	Total		White		Black		Hispanic*		< High school		4y college degree	
		BL	MR	BL	MR	BL	MR	BL	MR	BL	MR	BL	MR
Total preterm live births (percent, <37 wk gestation)	***11.4***	12.7	11.4	11.5	10.2	18.3	16.3	12.3	11.3	--	--	--	--
**Mental health**													
Suicide (age adjusted, per 100,000 population)	***10.2***	11.3	13.0	13.7	16.4	5.1	5.7	12.1	14.1	--	--	--	--
% adolescents with major depressive episodes (12–17y)	***7.5***	8.3	11.4	8.8	12.0	7.1	9.1	8.5	11.5	--	--	--	--
**Nutrition, physical activity & obesity**													
% adults meeting aerobic physical activity and muscle strengthening guidelines (age adjusted, ≥18y)	***20.1***	18.2	21.4	20.7	23.3	14.8	19.7	11.3	16.6	5.2	7.9	25.5	29.3
% obesity among adults (age adjusted, 20+ y)	***30.5***	33.9	37.7	32.7	36.4	44.6	48.4	36.8	46.4	37.7	42.4	25.4	28.4
% obesity among children and adolescents (2–17y)	***14.5***	16.1	17.2	14.0	15.3	20.6	18.8	21.7	21.8	--	--	--	--
Mean daily intake of total vegetables (age adjusted, cup equivalents/1,000 kcal, 2+ y)	***1.16***	0.76	0.77	0.75	0.77	0.68	0.66	0.78	0.79	0.81	0.81	0.94	1.00
**Oral health**													
% persons who visited the dentist in the past year (age adjusted, 2+ y)	***49.0***	44.5	42.1	50.7	47.8	31.1	30.3	29.2	29.8	19.7	18.2	--	--
**Reproductive & sexual health**													
% sexually active females receiving reproductive health services in the past 12 mos (15–44 y)	***86.5***	78.6	77.3	79.8	76.1	83.8	84.8	72.1	77.5	66.5	69.8	81.2	81.2
% HIV-positive persons with knowledge of serostatus (13+ y)	***90.0***	80.9	87.0	83.7	89.2	79.9	86.7	79.3	84.7	--	--	--	--
**Social determinants**													
% students awarded a high school diploma 4 y after starting 9th grade	***87.0***	79.0	82.0	84.0	87.0	67.0	72.0	71.0	76.0	--	--	--	--

Indicator	Target	Total		White		Black		Hispanic*		< High school		4y college degree	
		BL	MR	BL	MR	BL	MR	BL	MR	BL	MR	BL	MR
**Substance abuse**													
% adolescents using alcohol or illicit drugs in past 30 days (12–17 y)	***16.6***	18.4	16.0	20.0	16.8	14.5	14.9	18.5	16.5	--	--	--	--
% adults binge drinking in past 30 days (18+ y)	***24.4***	27.1	27.0	27.9	27.5	24.9	25.3	29.7	29.6	22.0	23.1	25.6	26.1
**Tobacco**													
% adult cigarette smoking (age adjusted 18+ y)	***12.0***	20.6	15.3	22.6	17.4	20.7	16.6	21.6	16.5	29.8	25.6	10.2	7.3
% adolescent cigarette smoking in past month (grades 9–12)	***16.0***	19.5	10.8	22.5	12.4	9.5	6.5	18.0	9.2	--	--	--	--

Source: Healthy People 2020 Topics and Objectives (https://www.healthypeople.gov/2020/topics-objectives)

*For some indicators, only data for Mexican Americans was available.

-- data not provided on HP2020 website.
