# Supplementary material for: Health disparities monitoring in the U.S.: lessons for monitoring efforts in Israel and other countries
Source: Isr J Health Policy Res. 2018 Feb 28;7:14. doi: 10.1186/s13584-018-0208-1 (PMC6389259; doi:10.1186/s13584-018-0208-1)
Supplement: Supplementary file 3 — Table S3. Summary of inequality parameters and health indicators in key health disparities monitoring initiatives in the U.S. (PDF 446 kb) [file 13584_2018_208_MOESM3_ESM.pdf]

Supplemental Table 3. Summary of inequality parameters and health indicators in key health disparities monitoring initiatives in the U.S.

Inequality parameter or Indicator/Outcome	CDC Health disparities report 2013	Healthy People 2020 (Targets)	AHRQ Disparities Reports 2015
**INEQUALITY PARAMETERS**			
Race	x	x	x
Ethnicity	x	x	x
Income/economic status[1]			
Income-to-poverty ratio	x		
Per-capita income	x		
Annual household income	x	x	
Area income (by zip codes)	x		x
Employment status	x		
Poverty/income status (according to Federal Poverty Level)	x	x	x
Educational attainment	x	x	
Geographic location			x
U.S. census region	x		
Area of residence (urban/rural)	x	x	
Sex	x	x	
Sexual orientation/ identity	x	x	
Age	x	x	
Disability	x	x	
Country of birth	x	x	
Primary language	x		
**HEALTH INDICATOR/OUTCOME**			
Mortality			
Coronary and stroke deaths	x	x	
Drug-induced deaths	x		
Life Expectancy			
Life expectancy at birth	x	x	
Life expectancy at age 65		x	
Healthy Life Expectancy			
Expected years of life in good or better health		x	
Expected years of life free of limitation of activity	x	x	
Expected years of life free of selected chronic diseases		x	
Years of Potential Life Lost (YPLL)			
(In U.S., people who die before 75y)			
By all-cause mortality		x	

Inequality parameter or Indicator/Outcome	CDC Health disparities report 2013	Healthy People 2020 (Targets)	AHRQ Disparities Reports 2015
By cause-specific mortality		x	
Physically Unhealthy Days			
No. days in last 30 days self-rated physical health not good	x	x	
Mentally Unhealthy Days			
No. days in last 30 days self-rated mental health not good	x	x	
Self-Assessed Health Status	x		
Activities of daily living (self-care)		x	
Instrumental activities of daily living		x	
Play, school, or work		x	
Remembering		x	
Any other activity that they cannot do because of limitations caused by physical, mental, or emotional problems		x	
Chronic Disease Prevalence			
Cardiovascular disease		x	
Arthritis		x	
Hypertension	x		
Diabetes	x	x	
Asthma	x	x	
Cancer	x	x	
Chronic obstructive pulmonary disease (COPD)		x	
HIV infection	x		
Periodontitis among adults aged ≥30 y	x		
Health-related quality of life and well-being			
Patient reported outcomes measurement information systems (PROMIS) global health measure (global physical, mental, and social HRQoL through questions on self-rated health, physical HRQoL, mental HRQoL, fatigue, pain, emotional distress, social activities, and roles)	x	x	
Well-being measures (assess the positive evaluations of people's daily lives—when they feel very healthy and satisfied or content with life, the quality of their relationships, their positive emotions, their resilience, and the realization of their potential)		x	
Participation measures (individuals' assessments of the impact of their health on their social participation within their current environment. The principle behind participation measures is that a person with a functional limitation—for example, vision loss, mobility difficulty, or intellectual disability—can live a long and productive life and enjoy a good quality of life.)		x	

Inequality parameter or Indicator/Outcome	CDC Health disparities report 2013	Healthy People 2020 (Targets)	AHRQ Disparities Reports 2015
Access to health services			
Persons (%, <65y) with health insurance	x	x (100%)	x
Persons with usual primary care provider		x (83.9%)	x
Persons of all ages who have specific source of ongoing care		x (95.0%)	
Persons who did not receive or delayed getting medical care due to cost in the past 12 months (for AHRQ & HP2020, includes dental care and prescription medications; CDC dental care)	x	x (9.0%)	x
Persons under age 65 whose family's health insurance premiums and out-of-pocket medical expenses were more than 10% of total family income			x
Persons who report difficulty seeing a specialist			
Persons who reported that they experienced good communication with their health care provider (AHRQ: having open discussion, gathering information, understanding patient's perspective, sharing information, reaching agreement on problems and plans, providing closure)		x	x
Access to care (e.g., having health insurance, usual source of care, difficulties when seeking and receiving care as soon as wanted, workforce capacity, trauma center utilization)			x
Healthy living (maternal and child healthcare, lifestyle modification, clinical preventive services, functional status preservation and rehabilitation, supportive and palliative care)			x
Inefficient care due to use of services associated with more harm than benefit (ruptured appendix per 1,000 adult admissions with appendicitis; men aged 40+ who had screen prostate-specific antigen test in past year)			x
Rate of potentially preventable hospitalizations	x		x
Care coordination (transitions of care, integration of medication information, use of electronic health records)			x
Patient safety in hospitals, nursing homes, home health, ambulatory care, and infrastructure (in all settings)			x
Clinical preventive services			
Adults receiving colorectal cancer screening based on most recent guidelines (age adjusted, %, 50–75 y)	x	x (70.5%)	
Adults with hypertension with controlled blood pressure (age-adjusted, %, ≥18y)	x	x (61.2%)	

Inequality parameter or Indicator/Outcome	CDC Health disparities report 2013	Healthy People 2020 (Targets)	AHRQ Disparities Reports 2015
Persons with diagnosed diabetes whose A1c value is >9% (age-adjusted, %, ≥18y)		x (16.1%)	
Fully immunized young children (%, aged 19–35 mos.)		x (80.8%)	
% people receiving flu vaccine in last 12 mos.	x		
Breast cancer screening, diagnosis and treatment			x
Environmental quality			
Air Quality Index (AQI) exceeding 100 (number of billion person days, weighted by population and Air Quality Index value)		x (1.980)	
Children exposed to secondhand smoke (%; nonsmokers, 3–11y)		x (47.0%)	
Residential proximity to major highways	x		
Injury and Violence			
Fatal injuries (age adjusted, per 100,000 population)		x (53.7)	
Nonfatal work-related injuries/illnesses	x		
Fatal work-related injuries/illnesses	x		
Homicides (age adjusted, per 100,000 population)	x	x (5.5)	
Motor vehicle-related deaths	x		
Maternal, Infant, and Child Health			
Infant deaths (per 1,000 live births, <1y)	x	x (6.0)	
Total preterm live births (percent, <37 wk gestation)	x	x (11.4%)	
Mental Health			
Suicide (age adjusted, per 100,000 population)	x	x (10.2)	
Adolescents with major depressive episodes (%, 12–17y)		x (7.5%)	
Nutrition, Physical Activity, and Obesity			
Adults meeting aerobic physical activity and muscle strengthening federal guidelines (age adjusted, %, ≥18y)		x (20.1%)	
Obesity among adults (age adjusted, %, 18/20+ y)	x	x (30.5%)	
Obesity among children and adolescents (%, 2–17/19 y)	x	x (14.5%)	
Mean daily intake of total vegetables (age adjusted, cup equivalents per 1,000 calories, 2+ y)		x (1.1)	
Oral Health			
Persons who visited the dentist in the past year (age adjusted, %, 2+ y)		x (49.0%)	
Reproductive and Sexual Health			
Pregnancy and childbirth among females aged 10-19y	x		
Sexually active females receiving reproductive health services in the past 12 mos (%, 15–44 y)		x (86.5%)	
Knowledge of serostatus among HIV-positive persons (%,		x (90.0%)	

Inequality parameter or Indicator/Outcome	CDC Health disparities report 2013	Healthy People 2020 (Targets)	AHRQ Disparities Reports 2015
13+ y)			
Social Determinants			
Poverty	x	x	
Unemployment	x		
Employed in high-risk injury and illness occupations	x		
Children aged 0-17 years living with ≥1 parent employed year round, full time		x	
Proportion of households that spend >30% or >50% of income on housing		x	
Food insecurity among households/children		x	
Students awarded a high school diploma 4 years after starting 9th grade (%)/adults who have completed high school	x	x (82.4%)	
Proportion of 4th grade students reading at or above the proficient achievement level for their grade		x	
High school completers enrolled in college the following fall		x	
Proportion of children with disabilities, aged 0-24 mos, who receive early intervention services in home or community-based settings		X (95.0%)	
Proportion of parents who read to their young child		X (52.6%)	
Neighborhood and built environment (5 objectives related to reducing crime and violence)		x	
Proportion of occupied housing units with moderate or severe physical problems		X (4.2%)	
Blood lead level in children aged 1-5y		x	
Minimize risks to human health and environment from hazardous sites		x	
Census tract without at least 1 healthier food retailer[2]	x		
No. states with state-level policies incentivizing food retail outlets to provide foods encouraged by the Dietary Guidelines for Americans		X (18)	
Proportion of children who ever lived with a parent who served time in jail/prison		x	
Proportion of persons eligible to participate in elections who register and actually vote		x	
Substance Abuse			
Adolescents using alcohol or illicit drugs in past 30 days (%, 12–17 y)		x (16.6%)	
Binge drinking in past 30 days—Adults (%,	x	x (24.4%)	

Inequality parameter or Indicator/Outcome	CDC Health disparities report 2013	Healthy People 2020 (Targets)	AHRQ Disparities Reports 2015
18+ y)			
Tobacco			
Adult cigarette smoking (age adjusted, %, 18+ y)	x	x (12.0%)	
Adolescent cigarette smoking (%, grades 9–12)	x	x (16.0%)	
HEALTH SERVICES INFRASTRUCTURE/WORKFORCE			
% clinicians receiving scholarships and loan repayment services			
% of degrees awarded to health professionals, para-professionals			
% of practicing physicians, nurses, dentists			
Racial and ethnic composition of health care workforce			
Racial and ethnic composition of primary care medical residents			x
GENERAL POPULATION HEALTH			
% low birthweight infants			
Tuberculosis	x		

black font-commonly available data, gray font-rarely available data

[1]The SES parameter used in the CDC report varied from one health indicator to another, and depended upon the SES data available for the specific health indicator.

[2]A healthier food retailer is one that sells the various foods recommended in the *Dietary Guidelines for Americans*, including fruits and vegetables, whole-grain foods, and low-fat milk.
