# Supplementary material for: Health disparities monitoring in the U.S.: lessons for monitoring efforts in Israel and other countries
Source: Isr J Health Policy Res. 2018 Feb 28;7:14. doi: 10.1186/s13584-018-0208-1 (PMC6389259; doi:10.1186/s13584-018-0208-1)
Supplement: Supplementary file 4 — Table S4. Healthy People 2010 Leading Health Indicators by inequality parameters. (PDF 1379 kb) [file 13584_2018_208_MOESM4_ESM.pdf]

Supplemental Table 4. Healthy People 2010 Leading Health Indicators by inequality parameters

Figure LHI-2. Health Disparities Table for Leading Health Indicators

Disparities from the best group rate for each characteristic at the most recent data point and changes in disparity from the baseline to the most recent data point.

	Race and Ethnicity								Sex		Education				Income				Location		Disability	
Population-based objective	American Indian or Alaska Native	Asian	Native Hawaiian or Other Pacific Islander	Two or more races	Hispanic or Latino	Black, not Hispanic	White, not Hispanic	*Summary index*	Female	Male	Less than high school	High school graduate	At least some college	*Summary index*	Poor	Near poor	Middle/high income	*Summary index*	Urban or metropolitan	Rural or nonmetropolitan	Persons with disabilities	Persons without disabilities
**Physical Activity**																						
22-2. Regular physical activity—Moderate or vigorous (age adjusted, 18+ years) (1997, 2008)[1]*							B[i]			B			B						B			B
22-7. Vigorous physical activity in students (grades 9–12) (1999, 2009)*							B			B												
**Overweight and Obesity**																						
19-2. Obesity in adults (age adjusted, 20+ years) (1988–94, 2005–08)[2,3]*					↓[ii]		B		↓	B					↓		B					B
19-3c. Obesity in children and adolescents 6–19 years (1988–94, 2005–08)[3]*					ii	B[i]	b		B								B					
**Tobacco Use**																						
27-1a. Cigarette use by adults (age adjusted, 18+ years) (1998, 2008)[1]*		b			B				B			↑	B		↑		B	↑			↑	B
27-2b. Cigarette use in past month by students (grades 9–12) (1999, 2009)*						B[i]	↑↑↑	iii	B													
**Substance Abuse**																						
26-10a. Adolescents not using alcohol or illicit drugs in past 30 days (12–17 years) (2002, 2008)[4]*		b				B				B					B[i]							
26-10c. Adults using illicit drugs in past 30 days (18+ years) (2002, 2008)*		b			B				B				B									
26-11c. Adults binge drinking in the past month (18+ years) (2002, 2008)[4]*		b				B			B							B						
**Responsible Sexual Behavior**																						
13-6a. Condom use among sexually active unmarried persons—females (18–44 years) (1995, 2006–08)[5‡]					↑	B[i]		↑				↑	B				B		B	B[i]	iv	B
b. Condom use among sexually active unmarried persons—males (18–44 years) (2002, 2006–08)[5‡§]					↓	B	↓	↓			↓		B	↓	↓		B			B[i]		B
25-11a. Students who never had sexual intercourse (grades 9–12) (1999, 2009)‡							B		B	B[i]												
25-11b. Students who had sexual intercourse, but not in the past 3 months (grades 9–12) (1999, 2009)‡§					B					B												
25-11c. Students who used condoms at last intercourse (grades 9–12) (1999, 2009)‡§					↓		B[i]	↓		B												

Source: National Center for Health Statistics. *Healthy People 2010 Final Review*. Hyattsville, MD. 2012.

Figure LHI-2. Health Disparities Table for Leading Health Indicators (continued)

Population-based objective	Race and Ethnicity: American Indian or Alaska Native	Asian	Native Hawaiian or Other Pacific Islander	Two or more races	Hispanic or Latino	Black, not Hispanic	White, not Hispanic	*Summary index*	Sex: Female	Male	Education: Less than high school	High school graduate	At least some college	*Summary index*	Income: Poor	Near poor	Middle/high income	*Summary index*	Location: Urban or metropolitan	Rural or nonmetropolitan	Disability: Persons with disabilities	Persons without disabilities
**Mental Health**																						
18-1. Suicide (age adjusted, per 100,000 population) (1999, 2007)[6]*§	↑	↑[v]			↑	B	↑↑	↑	B	↓↓		↑	B									
18-9b. Treatment for adults with depression (18+ years) (2002)*							B		B				B									
**Injury and Violence**																						
15-15a. Deaths from motor vehicle crashes (age adjusted, per 100,000 population) (1999, 2007)[6]*		B[v]					↑		B	↑	↑	↑	B	↑								
15-32. Homicides (age adjusted, per 100,000 population) (1999, 2007)[6]*		B[i,v]			↑	↑↑↑		↑↑	B	↑↑			B									
**Environmental Quality**																						
8-1a. Percent of persons exposed to ozone (1997, 2010)[7]†	B				↑				B	B									↑↑↑	B		
27-9. Exposure to tobacco smoke at home among children (≤6 years) (1994, 2005)[8]*§					b		B			B					↑↑↑	↑↑	B	↑↑				
27-10. Exposure to environmental tobacco smoke among nonsmokers (age adjusted, 4+ years) (1988–94, 2005–08)*					B[ii]	↑↑		↑	B		↑	↑	B	↑								
**Immunization**																						
14-24a. Fully immunized young children 19–35 months (1998, 2008)[9,10]*	b	b		B[i]					B[i]	B						B						
14-29a. Vaccination of noninstitutionalized high-risk older adults—Influenza vaccine in past 12 months (age adjusted, 65+ years) (1998, 2008)[1]*							B	iii	B[i]				B								B	
b. Vaccination of noninstitutionalized high-risk older adults—Pneumococcal vaccine ever received (age adjusted, 65+ years) (1998, 2008)[1]*					↑		B	↑	B[i]	↑			B								B	
**Access to Health Care**																						
1-1. Persons with health insurance (<65 years) (1997, 2008)[1]*	↓↓						B	iii	B						↓↓	↓	B	↓↓	B		B	
1-4a. Source of ongoing care (1998, 2008)[1]*							B	iii	B							↑	B	↑		B	B	
1-9a. Hospitalization for pediatric asthma (admissions per 10,000 population, <18 years) (1996, 2008)*§									B													
16-6a. Prenatal care beginning in first trimester (1998, 2002)*	↑	v					B				↑	↑	B	↑								

Figure LHI-2. Health Disparities Table for Leading Health Indicators (continued)

NOTES

See DATA2010 at http://wonder.cdc.gov/data2010 for all Healthy People 2010 tracking data.

Years in parentheses represent the baseline and most recent data years (if available).

Disparity from the best group rate is defined as the percent difference between the best group rate and each of the other group rates for a characteristic (e.g., race and ethnicity). The summary index is the average of these percent differences for a characteristic. Change in disparity is estimated by subtracting the disparity at baseline from the disparity at the most recent data point. Change in the summary index is estimated by subtracting the summary index at baseline from the summary index at the most recent data point. See Technical Appendix for more information.

LEGEND

**The "best" group rate** at the most recent data point.	B: The group with the best rate for specified characteristic.	b: Most favorable group rate for specified characteristic, but reliability criterion not met.	Reliability criterion for best group rate not met, or data available for only one group.	
		**Percent difference from the best group rate**		
**Disparity from the best group rate** at the most recent data point.	Less than 10%, or difference not statistically significant (when estimates of variability are available).	10%–49%	50%–99%	100% or more
**Changes in disparity** over time are shown when: (a) disparities data are available at both baseline and most recent time points; (b) data are not for the group(s) indicated by "B" or "b" at either time point; and (c) the change is greater than or equal to 10 percentage points and statistically significant, or when the change is greater than or equal to 10 percentage points and estimates of variability were not available. See Technical Appendix.		**Increase** in disparity (percentage points)		
		↑ 10–49 points	↑↑ 50–99 points	↑↑↑ 100 points or more
		**Decrease** in disparity (percentage points)		
		↓ 10–49 points	↓↓ 50–99 points	↓↓↓ 100 points or more
**Availability of Data**		Data not available.	Characteristic not selected for this objective.	

FOOTNOTES

* Measures of variability were available. Thus, the variability of best group rates was assessed, and statistical significance was tested. Disparities of 10% or more are displayed when the differences from the best group rate are statistically significant at the 0.05 level. Changes in disparities over time are indicated by arrows when the changes are greater than or equal to 10 percentage points and are statistically significant at the 0.05 level. See Technical Appendix.

† Measures of variability were not available. Thus, the variability of best group rates was not assessed, and statistical significance could not be tested. Nonetheless, disparities and changes in disparities over time are displayed according to their magnitude. See Technical Appendix.

‡ Measures of variability were available only for the most recent data. Thus, the variability of best group rates was assessed only for the most recent data, and statistical significance was tested only for the most recent data. Disparities of 10% or more are displayed when the differences from the best group rate are statistically significant at the 0.05 level. Changes in disparities over time are displayed according to their magnitude, since measures of variability were not available at baseline and therefore statistical significance of changes in disparity could not be tested. See Technical Appendix.

§ Supplemental measure. See LHI chapter text for more information.

1 Baseline data by race and ethnicity are for 1999.

2 Baseline data by disability status are for 1991–94.

3 Data by income are categorized using only two groups: lower income (≤130% of Federal poverty level, displayed under "poor") and higher income (>130% of Federal poverty level, displayed under "middle/high income").

4 Baseline data by income are for 2005.

5 Data by education level are for persons aged 25–44 years.

6 Most recent data by education level are for 2002.

7 Most recent data by race and ethnicity, by sex, and by location, are for 2004.

8 Baseline data by race and ethnicity are for 2005.

9 Baseline data by race and ethnicity are for 2000.

10 Baseline data by income exclude "middle/high income" for comparability with most recent data year.
